# A short-lived fish with long-lasting effects: hallmarks of aging in *Nothobranchius furzeri*

**DOI:** 10.3389/fragi.2025.1741819

**Published:** 2026-01-14

**Authors:** Oksana Savel, Julien Lehmann, Yusuf Kaan Poyraz, Melissa Marie Page

**Affiliations:** Louvain Institute of Biomolecular Science and Technology, UCLouvain, Louvain-la-Neuve, Belgium

**Keywords:** aging hallmarks, healthspan, killifish, lifespan, *N. furzeri*

## Abstract

Our world is facing a global aging crisis with an increasing number of people living longer in poor health, as indicated by a gap between lifespan and healthspan. It is necessary to improve our knowledge of the biomolecular and cellular pathways implicated in aging to improve the overall healthspan of the population and lift the economic and social burden of age-related diseases. Gerontologists have defined twelve hallmarks of aging to study them efficiently. Here we review each aging hallmark in the context of *N. furzeri*, a short-lived model fish. Introduced to the lab in 2003, this fish has the shortest vertebrate lifespan recorded in captivity. Depending on the strain, it lives between 2 months to 1 year. While aging, it develops several age-related phenotypes experienced by humans, such as emaciation, spine curvature, locomotor and cognitive defects. We summarize that aged *Nothobranchius furzeri* develop characteristics of each hallmark with high similarity to humans and other aging models. For several of these hallmarks, interventions that accelerate aging clearly leads to reduced health and a shorter lifespan, expanding our knowledge on molecular mechanisms favoring shorter healthspan. Interventions that decelerate aging have demonstrated a positive impact on health or an extension to lifespan, that could be transferred to humans for an increased healthspan. For example, the link between glucose metabolism and ER stress or the use of young microbial gut transplant to improve health are two discoveries made in *N. furzeri* and are of relevant importance for human healthy aging. By comparing similar ages and strains and by using standardized breeding procedures, the *N. furzeri* community will continue to greatly contribute to aging research. Creating stable transgenic lines and finding a way to administer drugs efficiently are two challenges that must be addressed to test novel targets of interests or therapies in each hallmark of aging.

## 1 Introduction

The global number of people aged 60 years and older continues to increase. In 2019, it was estimated that 1 billion people fit into this category and by 2050 this number is predicted to reach 2.1 billion (WHO). The rise in life expectancy has been largely driven by ameliorations to public health and medicine. While an increase in lifespan seems desirable; the time spent in good health, referred to as healthspan, unfortunately does not often follow the same trend, with individuals spending several final years living in poor health (Garmany and Terzic, 2024). This highlights the importance of research dedicated to understanding the molecular and cellular pathways that are implicated in aging with the goal of prevention and treatment of age-related diseases and to extend human healthspan.

To better understand the underlying mechanisms of aging, López-Otín and colleagues published two important reviews that together propose twelve hallmarks of aging; genomic instability, telomere attrition, epigenetic alterations, loss of proteostasis, disabled autophagy, deregulated nutrient-sensing, mitochondrial dysfunction, cellular senescence, stem cell exhaustion, altered intercellular communication, chronic inflammation and, dysbiosis (López-Otín et al., 2013; López-Otín et al., 2023). To be classified as a hallmark, the process must 1) manifest with normal age, 2) increase the rate of aging when experimentally increased, and 3) decrease or reverse the rate of aging when experimentally reduced. While focusing on human aging, these two reviews highlighted scientific knowledge gained from the use of model organisms, including *Nothobranchius furzeri* (turquoise killifish) a typical hobby fish that was introduced to the lab in 2003 and which has the shortest vertebrate lifespan recorded in captivity (Valdesalici and Cellerino, 2003).

Turquoise killifish are naturally found throughout Mozambique and Zimbabwe. They live in temporary ponds during the rainy season where they deposit their eggs in the muddy soil. During the dry season, unhatched eggs enter diapause until the return of the rainy season, which signals their reentry into the developmental cycle. The annual change and the length of seasons offer one explanation for the short lifespan that ranges between 2 months to 1 year depending on the collection location, which defines the different lab strains. First collected in 1969 from the Gonarezhou National Park, the GRZ-AD strain has become a highly inbred lab strain (Jubb, 1971; Vanhunsel et al., 2021; Terzibasi et al., 2007). Often simply referred to as GRZ; it is the shortest-lived strain with a lifespan ranging from 2 to 9 months. Several fish have been collected from Mozambique from 2004 onwards and include the experimentally used strains MZM-0403, MZM-0410, MZM-0703, MZCS-222 and MZM1001. These strains are often referred to as wild derived strains, and their lifespan ranges between 4 months to 1 year (Terzibasi et al., 2008).

In addition to a condensed lifespan, aged *N. furzeri* present several human age-related phenotypes, such as emaciation, spine curvature, and cognitive and locomotor defects (Terzibasi et al., 2008; Genade et al., 2005). While these fish were included in the characterization of one aging hallmark by López-Otín et al. (2023), their growing use in research highlights their worth in the continued study of aging hallmarks. While several reviews have been published on the use of *N. furzeri* as models of aging, they primarily focus on specific hallmarks (Morabito et al., 2024; Raggio et al., 2025) or specific tissues (de Bakker and Valenzano, 2023). With this review, we will summarize the hallmarks of aging that have been identified across several tissues within *N. furzeri* and comment on how this model can be used to address ongoing aging research. We will discuss the various parameters that need to reach a consensus within the *N. furzeri* community. For example, it is necessary to highlight and unify the choice of strains, choice of ages, dietary procedures, availability of genetic tools, administration of drugs. Without this consensus, the multiplicity of existing parameters in published studies complicates comparisons and affects the progress made in aging research using *N. furzeri* as a model. For simplicity, throughout the review we use the terms young, middle-aged, and old when referring to the ages selected for individual studies; however, we direct the reader to verify the exact ages as they may differ between the original studies. While several genetic engineering protocols have been published using the *N. furzeri*, not all have included lifespan studies (Oginuma et al., 2022; Harel et al., 2015; Harel et al., 2016; Hartmann and Englert, 2012; Allard et al., 2013; Valenzano et al., 2011; Rozenberg et al., 2023). Therefore, when possible, we include if interventions, genetic or otherwise have been used to explore the rate of aging in *N. furzeri* for each hallmark (Table 1).

**TABLE 1:** Interventions impacting health and aging rate of *Nothobranchius furzeri*. “Negative impact on health”, implies that the method used led to an aging phenotype in young fish or led to deleterious health effects in aged fish. If unspecified, either both sexes were used or it was unreported by the authors.

Hallmark of aging	Experimental accentuation	Effect of experimental accentuation	Therapeutic intervention	Effect of therapeutic intervention
Genomic instability	Not tested	☐ Decrease lifespan ☐ Negative impact on health	Not tested	☐ Increase lifespan ☐ Positive impact on health
Telomere attrition	*Tert* KO line (Harel et al., 2015)	✗ Decrease lifespan: not reported ✓ Negative impact on health	Not tested	☐ Increase lifespan ☐ Positive impact on health
Epigenetic alterations	Not tested	☐ Decrease lifespan ☐ Negative impact on health	Not tested	☐ Increase lifespan ☐ Positive impact on health
Loss of proteostasis	Bortezomib (intraperitoneal injection) (Kelmer Sacramento et al., 2020; Di Fraia et al., 2025)	✗ Decrease lifespan: not reported ✓ Negative impact on health	*α-synuclein* KO line (Matsui et al., 2019)	✗ Increase lifespan ✓ Positive impact on health
			*appa* KO line (preprint Bakker et al., 2024)	✗ Increase lifespan: not reported ✓ Positive impact on health
			Tunicamycin (aqueous exposition – Xbp1-GFP reporter line) (Semmy et al., 2025)	✗ Increase lifespan: not reported ✓ Positive impact on health
Disabled autophagy	*Atg5* KO line (Harel et al., 2015)	Not reported	Not tested	☐ Increase lifespan ☐ Positive impact on health
Deregulated nutrient-sensing	Not tested	☐ Decrease lifespan ☐ Negative impact on health	*Dnd* KO males^a^ (Moses et al., 2024) *Dnd* KD males^a^ (Abe et al., 2024) Caloric restriction (Terzibasi et al., 2009) *Aprt* heterozygous males (Astre et al., 2023) AMPKγ1 constitutively activated line (Ripa et al., 2023a) *C/EBPα* KO males (Müller et al., 2025)	✓ Increase lifespan ✓ Positive impact on health ✓ Increase lifespan ✓ Positive impact on health ✓ Increase lifespan ✓ Positive impact on health ✓ Increase lifespan ✗ Positive impact on health ✓ Increase lifespan ✓ Positive impact on health ✓ Increase lifespan ✓ Positive impact on health
Mitochondrial dysfunction	High-dose rotenone (water treatment) (Baumgart et al., 2016)	✓ Decrease lifespan ✗ Negative impact on health: not reported	Low-dose rotenone (water treatment) (Baumgart et al., 2016)	✓ Increase lifespan ✗ Positive impact on health: not reported
Cellular senescence	Not tested	☐ Decrease lifespan ☐ Negative impact on health	D + Q treatment (peritoneal injection-females only/oral gavage) (Ma et al., 2025; Van houcke et al., 2023)	✗ Increase lifespan: not reported ✓ Positive impact on health
Stem cell exhaustion	Not tested	☐ Decrease lifespan ☐ Negative impact on health	Not tested	☐ Increase lifespan ☐ Positive impact on health
Altered intercellular communication	Not tested	☐ Decrease lifespan ☐ Negative impact on health	Dapagliflozin (preprint Paulmann et al., 2025)	✗ Increase lifespan ✓ Positive impact on health
Chronic inflammation	*cGas* KO fish (preprint Ballhysa et al., 2024) *Sting* KO fish + irradiation (preprint Ballhysa et al., 2024)	✗ Decrease lifespan ✓ Negative impact on health ✗ Decrease lifespan: not reported ✓ Negative impact on health	D + Q injection after TBI (Van houcke et al., 2023)	✗ Increase lifespan: not reported ✓ Positive impact on health
Dysbiosis	Not tested	☐ Decrease lifespan ☐ Negative impact on health	Recolonization using gut microbiota of young fish (Smith et al., 2017)	✓ Increase lifespan ✓ Positive impact on health

^a^ Intervention that led to inverse effects in females.

### 1.1 Genomic instability

The genome of *N. furzeri* (GRZ) was sequenced 10 years ago with an estimated length between 1.3 and 2.2 Gb, encoding for a predicted 22,500 genes (Reichwald et al., 2015; Valenzano et al., 2015). Earlier this year, a genome assembly for MZM-0403 strain using third generation sequencing technology (PacBIO HiFI) was published with a length of 1.51 Gb and 24,060 protein-coding genes (Johnson et al., 2025). The species possesses 38 chromosomes (2n = 38). Mature male and female *N. furzeri* can be distinguished due to sexual dimorphism, however the presence of sex determining chromosomes (XX/XY system) allows researchers the possibility to address sex-linked diseases at earlier developmental stages, something that is lacking in zebrafish, another commonly used teleost to explore aging (Valenzano et al., 2009; Dohi and Matsui, 2022; Nagabhushana and Mishra, 2016).

#### 1.1.1 Nuclear DNA

Age-related accumulation of DNA mutations is well-documented in human tissues; believed to be due to an imbalance in the cost-benefit of DNA repair at advanced ages (Nik-Zainal and Hall, 2019; Gorbunova et al., 2007). For example, in short-lived species, an age-related decline in DNA repair could be explained by a trade-off between somatic and germline maintenance, although recent findings demonstrate some signs of reproductive senescence in *N. furzeri* (Chen et al., 2020; Cattelan and Valenzano, 2025). *Nothobranchius furzeri* reach sexual maturity within the rainy season and therefore spend energy on growth and reproductive pathways more so than on DNA repair mechanisms. Indeed, an age-related decrease in genes and/or proteins involved in DNA repair mechanisms has been reported in *N. furzeri*, indicating that genomic instability increases with advanced age in these fish. This noted reduction may be linked to the increased occurrence of neoplasms in the liver of older *N. furzeri* (Di Cicco et al., 2011; Baumgart et al., 2015), although histopathological identification has recently put these results into question (Dyková et al., 2021).

Age-dependent downregulation of genes related to the gene ontology (GO) biological process term “DNA replication”, and to several Kyoto Encyclopedia of Genes and Genomes (KEGG) pathways including “mismatch repair”, “nucleotide excision repair”, “homologous recombination”, and “base excision repair” have been reported from RNA-seq analysis of various *N. furzeri* tissues over several ages (Baumgart et al., 2014; Fumagalli et al., 2020; Cencioni et al., 2019). Genes linked to DNA replication were similarly found to be downregulated in aged skeletal muscle using RT-qPCR. In contrast, γ-H2A.X, a marker for double-strand DNA breaks, was enriched in the heart, visual system, skeletal muscle and head kidney isolated from old fish, indicating a negative correlation between DNA repair and damage (Vanhunsel et al., 2021; Cencioni et al., 2019; preprint Morabito et al., 2023, Ma et al., 2025). Furthermore, a longitudinal RNA-seq analysis of fin clips that were collected from MZM-0410 fish 10- and 20-week post hatching (wph) revealed an age-dependent downregulation of genes classified in such KEGG pathways as “DNA replication”, “nucleotide excision repair”, “base excision repair”, and “homologous recombination”. In addition to fin sampling, these fish were used to assess longevity; fish with shorter lifespans had more severe downregulations of the above pathways, indicating that these pathways can be used to assess *N. furzeri* mortality risk (Kelmer Sacramento et al., 2020). In a multi-species RNA-seq analysis (including zebrafish, MZM-0410 *N. furzeri*, mouse and human) that comprised sampling three tissues over five ages; the functional category “cell division”, containing several GO processes, such as “chromosome segregation”, “base excision repair”, “DNA replication initiation”, decreased with age in *N. furzeri* and humans. The GO term “DNA repair” was also reduced, which has been reported in several age-related diseases, highlighting the importance of DNA repair in both normal aging and age-related diseases (Aramillo Irizar et al., 2018).

Proteomic data revealed a similar age-related downregulation of diverse genomic stability processes. For example, proteins within the “base excision repair” pathway were reduced in old GRZ telencephalons (Van houcke et al., 2023). At both the proteome and transcriptome level, the KEGG pathway “mismatch” was reduced in old MZM-0410 brains, however in general the proteome and transcriptome were reported to decouple in old brains (Kelmer Sacramento et al., 2020). A decoupling was also reported in a separate study in which protein and transcript levels differed in the old brain of MZM-0410 *N. furzeri*; DNA repair and RNA-binding proteins were decreased whereas the transcript levels remained stable with age. DNA repair and RNA-binding proteins are important in transcription and translation processes. Therefore, their downregulation could affect both processes and provides a link between genomic instability and disrupted protein homeostasis (Di Fraia et al., 2025).

Phylogenetic studies identified several genes linked to genome stability that have been positively selected for within *N. furzeri.* These genes contain at least one site that is susceptible to mutation compared to other fish species (Valenzano et al., 2015). Comparing this list with known aging-related genes from human and mouse databases, Sirtuin6, which has been found to regulate lifespan in part due to genomic stability, was positively selected for within *N. furzeri* (Ma and Gladyshev, 2017; Tian et al., 2019; Roichman et al., 2021). Furthermore, the expression of *Sirt6* decreases in skeletal muscle and intestine with age in *N. furzeri* (Kabiljo et al., 2019). In addition, *Xrcc5*, a gene involved in DNA repair was also found under positive selection among several other species (Ma and Gladyshev, 2017). XRCC5 mutations have been linked to human mortality and knockout (KO) mice display signs of premature aging (Soerensen et al., 2012; Vogel et al., 1999). Therefore, mutations in XRCC5 may play a role in the short lifespan of *N. furzeri*.

#### 1.1.2 Mitochondrial DNA

The mitochondrial genome of *N. furzeri* has a size of 19,527 kb (Hartmann et al., 2011). This is notably longer than many other teleost fishes and mammals due to a duplication of the noncoding control region, however it is not an anomaly (Hartmann et al., 2011; Qian et al., 2018; Schirtzinger et al., 2012; Kim and Lee, 2004; Broughton and Dowling, 1994; Liu et al., 2018; Zhang et al., 2016). In avian species, the presence of an extended control region has been linked to longevity (Skujina et al., 2016). While this does not appear to be the case for *N. furzeri*, the duplication of the control region within *N. furzeri* mitochondrial DNA (mtDNA) may be of interest to explore potential mechanisms underlying aging. Contrary to mammalian aging, the common mtDNA deletion does not appear in *N. furzeri*, at least within skeletal muscle samples (Hartmann et al., 2011). DNA polymerase gamma (DNA polγ) is believed to be a driver for mtDNA deletions in humans, due to the high rate of mutations within the *Polg* gene (Lujan et al., 2020). While the gene expression pattern of mtDNA Polγ is decreased in adult GRZ skeletal muscle, the mutation rate of the *Polg* gene has not been measured, leaving open the possibility that this is a determining factor in the absence of mtDNA deletions (Cencioni et al., 2019).

#### 1.1.3 Nuclear architecture

*Lmna3*, encoding Lamin-A/C, has been identified as a gene involved in the aging process, and is under positive selection within *N. furzeri* (Valenzano et al., 2015)*.* In addition, the aggregation propensity of A-type nuclear lamins is increased in old *N. furzeri* hearts (Chen et al., 2024). Mutated human LMNA has been linked to both progeria syndrome and exceptional longevity (Eriksson et al., 2003; Conneely et al., 2012), therefore specific mutations selected throughout evolution and putative aggregation of *N. furzeri* lamin could be linked to its short lifespan. Apart from this data on lamins, no study has explored the relationship between nuclear lamina and lifespan in *N. furzeri*.

Single-cell preparations from several *N. furzeri* tissues have recently been published (Mariën et al., 2024; Teefy et al., 2023) and can be used to address the link between cell-specific regulation of genomes and aging and age-related diseases. Additionally, with the recent preprint detailing an epigenetic clock in *N. furzeri* (preprint Giannuzzi et al., 2024), we can soon expect to ask questions about how epigenetic modifications regulate genome stability.

### 1.2 Telomere attrition

Telomeres are G-rich repetitive non-coding DNA located at the end of chromosomes. Their length is shortened following every round of DNA replication due to the limitations of standard DNA polymerase in somatic tissues. In germline and stem cells, telomere length is maintained due to the activity of a telomerase enzyme composed of a catalytic protein subunit, TERT, and an RNA subunit, TERC (Vleck et al., 2003; Mason et al., 2024; Blackburn et al., 1989). A reduction in telomere length is observed with age in various organisms; however no clear correlation can be drawn between lifespan and telomere length. Rather, the rate at which telomeres are shortened correlate inversely with lifespan (Whittemore et al., 2019).

#### 1.2.1 Telomere length

Telomere length within GRZ and MZM-0403 strains have been measured in muscle and skin tissues and ranged in length between 4.5–6.7 kb, which is shorter than other select vertebrate models (i.e., mice, medaka fish, rats) (Hartmann et al., 2009). Telomere length was similar in young age of both strains however, with age telomere shortening was observed only in MZM-0403 fish. The unchanged telomere length during aging in GRZ fish contradicts findings that telomere shortening rate correlates negatively with lifespan (Whittemore et al., 2019). It is unclear if this result remains specific to this species or goes beyond to the genus of Nothobranchius, which includes approximately 100 other annual species. Biological sex can also contribute to telomere attrition, for example, *N. furzeri* males exhibit a shorter telomere length than females, along with a shorter lifespan (Reichard et al., 2022); a finding that aligns with telomere length in humans (Gardner et al., 2014). Sex-differences in telomere length may arise because certain telomere maintenance alleles are found on sex chromosomes (the “heterogametic sex disadvantage” hypothesis) (Barrett and Richardson, 2011). However, this phenomenon cannot explain similar telomere lengths in both sexes of many other species (Barrett and Richardson, 2011; Remot et al., 2020). Sex-dependent telomere length may also arise because males are more likely to compete for food, territory, and mates, and as such require higher metabolic costs and a higher number of cell divisions to increase growth rate (the “sex selection” hypothesis). As a result, this could produce a faster decline in telomere length in males (Young, 2018). A homozygous deletion of *Dnd1*, which is implicated in germline formation, resulted in lifespan extension and an age-related upregulation of telomere maintenance genes that is not observed in wildtype individuals (Moses et al., 2024). The increase in telomere maintenance genes could support the second hypothesis, and that these males may no longer compete for reproductive females (although this was not measured in the study) and therefore cellular energy is directed towards maintenance pathways.

#### 1.2.2 Telomerase activity

Expression and activity of telomerase was tested in various tissues of GRZ and MZM-0304 *N. furzeri* strains (Hartmann et al., 2009). *Tert* expression was higher in aged muscle and skin for MZM-0304 but not for GRZ. *Tert* and *Terc* expression and overall telomerase activity was variable between MZM-0304 tissues scoring higher for testis, gill, ovary followed by skin, spleen, eye, brain, liver and muscle. A *Tert* KO line of GRZ *N. furzeri* was generated to address potential links between telomere length and age-related phenotypes. While telomerase activity was undetectable, these fish appeared physically normal, albeit with reduced fertility due to atrophied reproductive organs at old age compared to WT fish (Harel et al., 2015). Unfortunately, the lifespan was not reported for these fish. Due to the tissue-specific levels of telomerase activity it would be interesting to determine whether this has a role in tissue-specific rates of aging, something that could be explored with the development of tissue-specific *Tert* KO lines (Bedbrook et al., 2023). At the same time, this could provide us with more information regarding how the non-telomeric activities of TERT may interact with other aging hallmarks, such as mitochondrial dysfunction, genome stability and stem cell exhaustion (Ségal-Bendirdjian and Geli, 2019).

### 1.3 Epigenetic alterations

Epigenetics refers to all modifications of gene expression that occur without altering the DNA sequence, and includes DNA methylation, histone modifications, chromatin remodeling, and non-coding RNA regulation (López-Otín et al., 2023). Over the past decades, epigenetic mechanisms have emerged as central regulators of aging (Booth and Brunet, 2016). Indeed, aging is associated with characteristic and progressive changes in the epigenome, often referred to as “epigenetic drift”. Such alterations contribute to the functional decline of cells and tissues as well as the development of age-related pathologies by disrupting gene regulatory networks and impairing genome stability.

#### 1.3.1 DNA methylation

DNA methylation refers to the addition of a methyl group at specific locations in the DNA, usually at the fifth carbon of cytosine (5mC) in a CpG dinucleotide context. During aging, the genome undergoes global hypomethylation accompanied by site-specific hypermethylation, which can alter gene expression and trigger genomic instability (Jones et al., 2015). In vertebrate genomes, methylation is established and maintained by the DNA methyltransferase (DNMT) family, with DNMT3A and DNMT3B mediating *de novo* methylation, and DNMT1 preserving methylation patterns during DNA replication. Demethylation occurs in response to the activity of Ten-Eleven Translocation (TET) family member enzymes which act by sequentially oxidizing the 5mC to 5-hydroxymethylcytosine (5hmC), 5-formylcytosine and 5-carboxylcytosine before the methyl group is removed by the thymine DNA glycosylase enzyme during the base excision repair process (Mattei et al., 2022).

Zupkovitz et al. (2021) revealed a tissue-specific DNA hypomethylation during aging in GRZ fish (Zupkovitz et al., 2021). A progressive decline in global 5mC levels was observed in liver and muscle tissues. In contrast, 5mC levels remained unchanged in the brain; however, the authors reported an age-dependent reduction in 5hmC within this tissue. These results were accompanied by transcriptional downregulation in some DNMTs and TETs which could indicate a deregulation of DNA methylation machinery over time (Zupkovitz et al., 2021).

RNA-seq analysis performed using the whole brain from MZM-0410 revealed a downregulation of *Dnmts* with advanced age. These changes were accompanied by transcriptional remodeling involving chromatin-associated genes, including components of the polycomb complex which is responsible for chromatin compaction and silencing genes through histone modification such as H3K27me3 (Baumgart et al., 2014). In a separate study, expression of *Dnmt1* and *Dnmt3a* was also reported to decrease with age in heart isolated from MZM-0410 *N. furzeri*. This repression was linked to the increased expression of the microRNA miR-29 family, which targets *Dnmt* transcripts. The data suggests that microRNA-mediated post-transcriptional control contributes to the age-dependent impairment of DNA methylation maintenance (Heid et al., 2017). This is corroborated by other articles highlighting the impact of miR-29 on *Dnmt* transcripts in cell lines (Fabbri et al., 2007; Morita et al., 2013). The causal impact of miR-29 on DNMT regulation has been directly demonstrated in zebrafish by using a transgenic line with inhibited miR-29 activity (Heid et al., 2017).

Based on discoveries related to DNA methylation and its close correlation with the aging process, epigenetic clocks have been developed as predictive aging models (Walther and Mann, 2011; Warner et al., 2024). These models use age-associated CpG methylation patterns to accurately predict chronological age and can be used to determine whether an individual is aging faster or slower than expected. Using reduced representation bisulfite sequencing and machine learning approaches, Giannuzzi *et al.* analyzed DNA methylation in the brain and caudal fin at different ages to develop the first epigenetic clock in MZM-0410 *N. furzeri* (preprint Giannuzzi et al., 2024). Chronological age was predicted within a median error of 1.5–3 weeks, depending on the model used. Furthermore, the clock designed for MZM-0410 fish was used to predict the chronological age of GRZ fish. It is important to now test whether the epigenetic clock can predict biological age in response to interventions that are known to increase or decrease the rate of aging.

#### 1.3.2 Transposable elements

Transposable elements (TE) are mobile repetitive genetic elements that are generally silenced in young tissues and de-repressed with age through DNA methylation. Several TE were found differentially expressed with age in brain, heart, muscle and spleen of GRZ fish (Xu et al., 2023). Compared to other tissues, in which TE were equally upregulated and downregulated, the brain was mainly characterised by an upregulation of TE. Subfamilies of TE (DNA transposons, LINE–long interspersed nuclear elements, LTR–long terminal repeat, SINE-short interspersed nuclear elements) were categorized and more changes were seen in LINE TE of the aging brains. De-repression of LINE has been linked to upregulation of inflammatory pathways, whereas high SINE expression has been associated with increased DNA repair pathways (Tsai et al., 2024). As DNA repair deficiencies occur with age in *N. furzeri* (see Genomic instability hallmark), this result supports the trade-off between reproduction and maintenance and a link between inflammaging and epigenetic modifications.

#### 1.3.3 Histone modifications

Histone modifications are epigenetic regulators that influence chromatin structure and gene expression. These include acetylation, methylation, phosphorylation, ubiquitination, and sumoylation. However, the former two are the most studied modifications in the context of aging (Kelly et al., 2010). Acetylation of lysine residues on histone tails, mediated by histone acetyltransferases (HATs), reduces the interaction between histones and DNA, leading to a more relaxed chromatin conformation which facilitates transcription. This modification is reversible through the action of histone deacetylases (HDACs), which promote chromatin condensation and gene silencing (Struhl, 1998). Histone methylation, in contrast, can either activate or repress transcription depending on the specific residue modified. These modifications are catalyzed by histone methyltransferases and removed by demethylases, contributing to dynamic and context-specific regulation of gene expression (Greer and Shi, 2012).

Transcriptomic analyses revealed downregulation of *Hdac1* and *Hdac3* across five ages in MZM-0410 (Baumgart et al., 2014). This reinforces data by Zupkovitz *et al.* where *Hdac1* expression was reported to consistently decline in brain, liver, and muscle tissues at both gene and protein levels during aging. *Hdac3* gene expression levels decreased across all tissues; in contrast, protein levels decreased with age only in the brain and muscle (Zupkovitz et al., 2018a). It is also important to note that HDACs possess non-histone modifying roles, and that gene and protein levels of HDACs may not directly be linked to histone modifications (Hess et al., 2022). However, using skeletal muscle, an increase in repressive histone marks (H3K27me3, H3K9me3, and H4K20me3), alongside a reduction in activating histone marks (H3K9ac and H4K16ac) was reported in aging MZM-0410 males. By comparing ChIP-seq and RNA-seq data an epigenetic regulatory effect of H3K27me3 and H3K9ac was highlighted. For example, the authors reported that downregulated genes showed age-related enrichment associated with H3K27me3 and a decrease in H3K9ac whereas upregulated genes showed the opposite pattern. These results suggest that aging in *N. furzeri* skeletal muscle shifts towards a more repressive chromatin state, silencing essential genes and contributing to functional decline and accelerated tissue aging (Cencioni et al., 2019).

#### 1.3.4 Chromatin remodeling

Chromatin remodeling is the dynamic reorganization of nucleosomes by ATP-dependent complexes to regulate DNA accessibility to transcription factors, RNA polymerase, or other epigenetic enzymes. Chromatin remodeling plays a key role in controlling gene expression and genome stability and works in concert with histone modifications (Ho and Crabtree, 2010).

In MZM-0410 *N. furzeri*, evidence for chromatin remodeling during aging primarily emerges through indirect markers and histone-based epigenetic modifications. For example, downregulation of DNMTs and chromatin regulators alongside upregulation of polycomb group components during aging were associated with increased levels of H3K27me3, suggesting enhanced chromatin compaction (Baumgart et al., 2014). Complementary transcriptomic analyses in MZM-0410 brain, liver, and skin tissues revealed age-associated differentially expressed genes distributed across the genome, suggesting a widespread deregulation of the chromatin state (Fumagalli et al., 2020).

#### 1.3.5 MicroRNA

MicroRNAs (miRNAs) are small non-coding RNAs that base-pair with complementary sequences of target mRNAs, leading to either mRNA degradation or inhibition of translation (Chuang and Jones, 2007). Depending on their activity, miRNAs have been linked to positive and negative lifespan regulation in invertebrates and appear to regulate tissue-specific processes in vertebrates (Kinser and Pincus, 2020).

In *N. furzeri*, miRNA expression is dynamically regulated during aging, with several age-associated miRNAs identified in brain tissues from both GRZ and MZM-0410 *N. furzeri*, however results following miRNA-seq analysis suggest that miRNA age-dependent regulation is not uniform across these two strains, at a similar biological age (50% survivorship). These differences may suggest that miRNA dynamics are influenced by genetic background and may potentially contribute to the strain-distinct aging rates and lifespans (Baumgart et al., 2012). The development of efficient CRISPR-Cas9 KO protocols in *N. furzeri* now offers the possibility to directly test whether age-associated miRNAs identified in transcriptomic experiments contribute to lifespan regulation (Rozenberg et al., 2023).

Together, these findings in *N. furzeri* highlight that aging is not governed by isolated epigenetic alterations, but rather by a deeply interconnected network of regulatory mechanisms. Changes in histone modification patterns correlate with shifts in chromatin architecture and accessibility. Chromatin remodeling further amplifies these effects, facilitating the establishment of a repressive epigenetic landscape that silences gene expression. The upregulation of polycomb components and the enrichment of H3K27me3 in aged tissues reflect this global reprogramming (Cencioni et al., 2019). Taken together, these different layers of epigenetic regulation interact functionally and reinforce one another, shaping the transcriptional and structural features of aging cells. The exceptionally short lifespan and rapid development of aging phenotypes in this species provides a unique opportunity to link molecular epigenetic alterations to the decline of the organism in a vertebrate context (preprint Giannuzzi et al., 2024; Zupkovitz et al., 2021; Zupkovitz et al., 2018b).

### 1.4 Loss of proteostasis

Protein homeostasis declines with age, resulting in incomplete, misfolded or erroneously translated proteins that can aggregate and/or fail to be delivered for repair by chaperones.

#### 1.4.1 Protein aggregation

The use of a non-specific aggregate marker indicated that proteins are more likely to aggregate in old brains of *N. furzeri* compared to young. Detection of ribosomal protein RPS6, suggest that the aggregates are associated with lysosomes, a finding also reported in the brains of old mice (Kelmer Sacramento et al., 2020). Several protein-specific aggregates have also been identified in aged *N. furzeri.* For example, α-synuclein inclusion bodies, a marker associated with Parkinson’s disease, were detected throughout the whole brain of old individuals while only detected in the medulla and spinal cord of young fish. This was accompanied by a decrease in the number of dopaminergic and noradrenergic neurons in the older brains. To determine whether α-synuclein had a role in the loss of neurons, homozygous fish deleted for α-synuclein were generated. While lifespan was not altered and no visible phenotype was reported, neuronal number remained constant throughout the lifespan of the KO fish, reinforcing that α-synuclein has a role in the observed neurodegeneration in WT fish (Matsui et al., 2019). TAR DNA-binding protein 43 kDA (TDP-43), whose aggregation is linked to amyotrophic lateral sclerosis and frontotemporal dementia, was found to co-localise with a non-specific protein aggregate stain in brain sections of old MZM-0410 brains, whereas no co-staining was detected in young brains (Louka et al., 2022). A form of amyloid beta, a peptide found in AD patients, accumulates in fish brains with age while *appa* (amyloid precursor protein a) KO fish experienced decreased cell death and inflammation, increased learning performance and a younger proteome (preprint Bakker et al., 2024). Together, these results highlight the feasibility of *N. furzeri* as models to explore brain aged-related pathologies.

Increased levels of SDS-resistant proteins recovered in high molecular weight protein fractions in aged GRZ brains suggest that protein aggregation positively correlates with age. Proteomic analysis of these fractions revealed a series of proteins with aggregation propensity and are predicted to have prion-like properties. For example, DDX5, which is an RNA helicase, forms aggregate-like puncta in *N. furzeri* brains sections. In young animals, puncta localized to nuclei whereas in old animals they were detected in nuclei and the cytosol. The role of DDX5 and other putative aggregating proteins identified during normal aging should be further explored during the progression of age-related neurodegenerative diseases (Harel et al., 2024). A comprehensive study exploring age-related protein aggregation using several *N. furzeri* tissues revealed a tissue-specific response and suggested that several unstudied aggregating proteins could be implicated in age-related diseases (Chen et al., 2024). For example, LMNA displays an increased aggregation propensity in the heart of old *N. furzeri.* As discussed above, while mutated copies of LMNA are detected in individuals diagnosed with forms of progeria, it is unclear if the aggregated proteins in the fish contain mutant homologs and/or the role for aggregated LMNA in the progeria disease (Worman, 2012).

#### 1.4.2 Chaperone activity

Collapse of proteostasis can be observed due to reduced chaperone activity. Age-related changes in the protein level of many chaperones have been reported in several *N. furzeri* tissues and reflect what has been published using other model organisms (Walther and Mann, 2011; Murgia et al., 2017; Golenhofen et al., 2004). Additionally, several actors of the chaperone-mediated autophagy, a specialized degradation process, are reduced in the old *N. furzeri* brain (Chen et al., 2024). Although chaperone activity has not been performed in *N. furzeri*, overall data suggests a decreased availability of chaperones with age. The size of the core chaperone network is decreased in the short-lived *N. furzeri* compared to the long-lived naked mole rat *H. glaber.* This finding along with low proteome aggregation reflects their fragile and robust protein homeostasis, respectively (Draceni and Pechmann, 2019).

Increased gene expression of chaperones specific to the inositol-requiring enzyme 1-X-box binding protein 1 (Ire1-Xbp1s) pathway of the ER unfolded stress response were found in old GRZ livers. This was accompanied by changes in ER shape and size, increased expression of *xbp1s* and several of its target genes. Together, these results revealed an activation of the ER stress response with age (Semmy et al., 2025). Interestingly, using a GFP construction to report Xbp1 activity, a downregulation of the Ire1-Xbp1s pathway was found in aged basal layer of the fish epidermis, where a decline in cell proliferation was observed. On the contrary, induction of Ire1-Xbp1s pathway by tunicamycin was able to activate cell proliferation in basal epidermal cells of treated fish, partially reversing the age-dependent loss of epidermal stem and progenitor cells and restoring the epidermis basal layer to a more youthful transcriptome. Glucose treatment similarly activated the Ire1-Xbp1s pathway to promote cell proliferation of the epidermis. Downregulation of glucose metabolism with advanced age may explain reduced activation of the Ire1-Xbp1s pathway and increased epidermal cell aging. The discovery of the glucose-Ire1-Xbp1s axis offers the potential to reveal new targets for helping healthy skin aging (Semmy et al., 2025).

#### 1.4.3 Proteasome expression

Central to loss of proteostasis is the proteasome; the 26S proteasome is composed of one catalytic subunit (20S) and one regulatory subunit (19S), and the 30S proteasome comprises two 19S subunits. In addition, the 20S subunit can function by itself (Sahu et al., 2021; STAR Protocols). RNA-seq analysis of whole MZM-0410 brains revealed a general age-related downregulation of genes coding for the proteasome, as well as reduced “protein folding” and “unfolding protein binding” GO terms (Baumgart et al., 2014). Unexpectedly, expression of proteasomal genes were upregulated within tissues collected from the oldest individuals. These results may reflect a compensatory mechanism in fish that faced extreme age, or the possibility that the oldest surviving fish experienced delayed aging. In any case, this finding has been similarly reported in fibroblasts derived from human centenarians (Chondrogianni et al., 2000). The proteasome was further explored by examining the balance between protein and transcript levels in whole brains at various ages (Kelmer Sacramento et al., 2020). A decrease in both proteasome protein and transcript levels was found in adult fish whereas for old fish, an imbalance was revealed. Particularly, the catalytic 20S proteasome, was upregulated at the protein level but not at the transcript level when comparing old and adult fish. Furthermore, activity levels of total 30S and 26S proteasome (chymotrypsin-like proteasome, CT-L) were downregulated after 5 wph.

CT-L proteasome activity can be experimentally reduced following an acute treatment of bortezomib. Intraperitoneal injections of bortezomib resulted in similar proteome changes between young treated and aged fish, indicating that the treatment signalled an aging phenotype that in part occurred through impaired proteostasis (Kelmer Sacramento et al., 2020). Chronic bortezomib treatment leading to 50% CT-L proteasome inactivation resulted in adaptative responses in protein levels (proteasome activators) and mRNA levels (autophagy genes *Atg5*, *Atg7*). This treatment also led to increased levels of specific chaperones, increased size of lysosomes and a reduction of mitochondrial content, all which have been identified in aged cells. However not everything was recapitulated: the observed decoupling between transcript and protein level of ribosomes and respiratory chain complexes occurred in opposite directions between aging and bortezomib treatment, suggesting that reduction in proteasome activity mimics some aging brain phenotypes but that others are due to different mechanisms (Di Fraia et al., 2025). Bortezomib treatment also led to an upregulation of autophagy-related proteins in brain samples. Therefore, further examination into the potential connection between the proteasome and autophagy may be warranted (Kelmer Sacramento et al., 2020). Although it may not fully explain aging, a decreased expression of proteasomal transcript was significantly correlated with an increase in mortality risk along lifespan in fish that were used in a longitudinal RNA-seq dataset of fin biopsies sampled at 10 and 20 wph (Kelmer Sacramento et al., 2020).

Increasing the activity of the proteasome and/or overexpressing chaperones increase lifespan in *M. musculus*, *Caenorhabditis elegans* and *D. melanogaster* (Bobkova et al., 2015; Anderson et al., 2022; Vos et al., 2016). The landscape of the proteasome, protein aggregates and chaperone levels have been well studied during normal aging of *N. furzeri.* While we could envision generating genetic fish lines to explore the overexpression or loss of specific players in proteostasis, we can also take advantage of cell culture protocols that have been put into place (Graf et al., 2013; Bagnoli et al., 2022; Součková et al., 2023) to explore whether specific protein damages have a stronger impact on the age-related decline in proteostasis.

### 1.5 Disabled autophagy

Autophagy is the process by which various cellular components are enclosed in vesicles or autophagosomes for lysosomal digestion. Manipulating the autophagy pathway has been shown to regulate aging in several organisms (Cassidy et al., 2020; Klionsky et al., 2021; Pyo et al., 2013; Lu et al., 2021).

#### 1.5.1 Autophagy-related gene expression

Transcriptomic and proteomic data from several *N. furzeri* aging studies reveal age-related changes linked to autophagy induction. Analysis of a longitudinal RNA-seq study using fins collected from a group of fish at 10 and 20 wph revealed “autophagy-related-genes” in the top 10 upregulated KEGG pathways (Kelmer Sacramento et al., 2020). While no specific research has been performed to study the mechanisms of autophagy during aging in *N. furzeri*, a starvation protocol (5 days of fasting) to induce autophagy in young GRZ and MZM-0403 *N. furzeri* highlighted the reactivity of several commercially available antibodies that could be used to explore this pathway further (Anagnostopoulos et al., 2021). A transgenic *N. furzeri* line for ATG5, which is necessary for vesicle formation, was also generated in 2015 but no further study using these fish has been published (Harel et al., 2015).

#### 1.5.2 Lysosomal degradation

Analysing the KEGG database, Baumgart *et al.* reported an age-related enrichment of genes within the whole brain belonging to pathways linked to “lysosome” (Baumgart et al., 2014). Enriched in this term were cathepsins, which are lysosomal hydrolases that have previously been linked to aging from a meta-analysis of rodent and human datasets (de Magalhães et al., 2009). An increased demand for lysosomal components may be in response to cellular waste that accumulates with age. For example, lipofuscin, composed of oxidized proteins and lipids found in lysosomes (Snyder and Crane, 2025), accumulates with age in liver and brain sections of GRZ and MZM-0410 *N. furzeri* (Terzibasi et al., 2008). In a separate cohort of fish, elevated expression levels of phagosome-related genes negatively correlated with lifespan at young age (Baumgart et al., 2016). It remains unclear as to what leads to this decoupling between expression levels related to the lysosome and phagosome, however this along with exploring how the interaction between autophagy and the exocytosis pathway are impacted during aging could be assessed using *N. furzeri*.

### 1.6 Deregulated nutrient-sensing

Nutrient-sensing pathways, which include mTOR, AMPK, insulin/insulin-like growth factor (IGF)-1 and sirtuins signalling cascades, detect and respond to available nutrients to regulate cellular metabolism, growth and homeostasis.

#### 1.6.1 Insulin/insulin-like growth factor-1 signalling

The insulin/IGF-1 signalling (IIS) pathway is highly conserved throughout evolution, regulating a metabolic switch between organismal growth and aging. Phosphoinositide 3-kinase (PI3K) and protein kinase B (Akt) are two kinases within the IIS cascade. PI3K-Akt enrichment has been linked to increased mortality risk in *N. furzeri*, supporting the knowledge that reduced IIS is linked to a longer lifespan (Kelmer Sacramento et al., 2020). When IIS is downregulated, forkhead transcription factors remain unphosphorylated and can be transported to the nucleus to upregulate the transcription of genes involved in longevity (Mathew et al., 2017). Several of the genes involved in IIS have been reported to be positively selected for in *N. furzeri*, however it remains to be determined whether this is due to their role in diapause (Mukhopadhyay et al., 2006; Hutfilz, 2022). Although there is an enrichment for binding sites of the transcription factors forkhead box A1 (FOXA1) and O3 (FOXO3) and restrictive element-1 silencing transcription factor (REST) during diapause, the length of diapause does not appear to affect adult lifespan in *N. furzeri* (Hu et al., 2020). Interested in how parental age could affect offspring growth levels, Api *et al.* measured target genes related to IIS in the offspring of MZM-0410 *N. furzeri* (Api et al., 2018). *Igf1* and *Insr* expression levels in young F1 females from old parents were reduced compared to F1 females hatched from young parents. In F1 males hatched from old parents, liver IGF binding protein-2 levels were significantly increased compared to F1 males from young parents. It would be beneficial to record the lifespan of the F1 generation to determine if these changes influenced the rate of aging (Api et al., 2018).

*Nothobranchius furzeri* were used in two independent studies to explore the trade-off between growth and longevity following germ cell ablation, using morpholino antisense oligonucleotides or CRISPR-Cas9 targeted against the germline-specific *Dnd* gene (Moses et al., 2024; Abe et al., 2024). Morpholino targeted knockdown of *Dnd* significantly increased median and mean lifespan in males. In addition, increased numbers of satellite cells, collagen levels and bone mineral density, indicated improved health in skeletal muscle, skin and bone, respectively in the knockdown males. In contrast, median and mean lifespan was significantly reduced by knockdown in females (Abe et al., 2024). The ablation resulted in an increased body length and mass in both sexes. Interested in the increased growth rate, the authors measured liver *Igf1* expression, which was significantly elevated in germ-cell ablated females. This was accompanied by increased reactivity for senescent-associated β-galactosidase (SA-β-Gal) compared to livers of control females. In contrast, liver *Igf1* levels in males remained similar between controls and germ-cell ablated individuals despite the difference in growth rate (Abe et al., 2024). CRISPR-Cas9 KO of *Dnd* significantly increased median and maximal lifespan in males, whereas there was no effect in female individuals compared to WT controls. Transcriptomic, metabolomic and histological analysis of the livers of old males suggested that germ cell ablation also improved overall health (Moses et al., 2024).

#### 1.6.2 Caloric restriction

The IIS pathway can be downregulated through dietary intervention, which extends lifespan and/or promotes signs of healthy aging in a wide range of animal models (López-Otín et al., 2013). Caloric restriction, as every other day feeding, extended median lifespan and 90th percentile lifespan within pooled female and male GRZ *N. furzeri* compared to *ad libitum* fed controls. While the same intervention resulted in a nonsignificant reduced median lifespan in wild-derived MZM-0410 fish, the 90th percentile lifespan was significantly increased. Caloric restriction, which was initiated in both strains at four wph, increased mortality until 15 wph in the MZM-0410 strain, resulting in a loss of 37% of this group (Terzibasi et al., 2009). Increased early mortality has similarly been reported in wild-derived mice challenged by caloric restriction, although the longest living wild-derived mice were those belonging to the group that were calorically restricted (Harper et al., 2006). Despite the differing effect on lifespan, caloric restriction reduced age-related lipofuscin accumulation, prevented behavioural decline, and reduced signs of age-related neurodegeneration in the telencephalon, optic tectum and hindbrain of both strains. In contrast, fish under caloric restriction had significantly higher levels of gliosis, indicating a dissociation between the markers used to detect neurodegeneration in response to caloric restriction (Terzibasi et al., 2009). Furthermore, gut transcriptomic analysis of fish challenged with an intermittent fasting protocol indicated a protective effect of fasting on several biological function (i.e., circadian clock, cell cycle, ribosome assembly, mitochondrial function, senescence, stem cell differentiation), as gene expression of old fasted animals were comparable to adult animals (Kothmayer et al., 2025). A recent study took advantage of in-house designed automated feeders to provide further insight into caloric and feeding-time restriction within the GRZ strain. This approach led to significantly reduced growth rates and longer lifespans in males and significantly reduced fecundity in female fish. These authors began to explore the impact of overfeeding; however, the effect on lifespan remains to be determined (McKay et al., 2022).

#### 1.6.3 AMPK pathway

*N. furzeri* have also been used to assess AMPK as an energy sensor and lifespan regulator. Due to difficulties faced in altering AMPK signalling through direct manipulation of the kinase, Astre *et al.* generated a heterozygous GRZ line with reduced levels of adenine phosphoribosyltransferase (APRT) (Astre et al., 2023). This gene is involved in AMP synthesis and as a result, allows for the manipulation of the nucleotide pool. Median and 90th percentile lifespan was significantly increased in male heterozygotes compared to wildtype controls. The lifespan extension in response to reduced APRT may share similar life-extending molecular mechanisms to lifelong intermittent fasting, for example, whereas median lifespan was significantly increased in both wildtype female and male fish subjected to intermittent fasting, there was no additional increase in lifespan within challenged heterozygous fish (Astre et al., 2023). The impact of fasting and refeeding was used to explore AMPK expression in visceral adipose tissue of young and old *N. furzeri* GRZ strain (Ripa et al., 2023a). A transcriptional change that was noted between the transition of fasting and refeeding in young fish was the differential expression of AMPKγ1 and γ2 subunits, which was not observed in old fish. In contrast, AMPKγ2 was highly expressed (gene and protein level) under both fed and fasted periods in the older fish. Compared to AMPKγ1, elevated AMPKγ2 activity has been linked to metabolic disorders in mice and humans [reviewed in Ripa et al. (2023a)], therefore the authors explored whether a constitutively active AMPKγ1 could protect against this phenotype. Fish with constitutively active AMPKγ1 had improved signs of metabolic health, as assessed by higher adipocyte turnover, reduced triglycerides, and reduced fasting blood glucose, and an increased lifespan, the latter being more pronounced in female fish (Ripa et al., 2023a).

CRISPR-Cas9 was used to disrupt the upstream open reading frame of C/EBPα, a transcription factor implicated in cell proliferation, differentiation, metabolism and senescence, in the ZMZ1001 strain of *N. furzeri*. This resulted in homozygous fish that do not express the inhibiting isoform of C/EBPα, which should positively impact the transcriptional activity of its other isoforms. Indeed, liver transcriptome analysis revealed upregulation of GO terms including several metabolic processes and enriched KEGG pathways included AMPK signaling pathway. While both sexes were used, increased lifespan and markers of improved healthspan were observed only in the KO males, suggesting that C/EBPα is implicated in nutrient and stress signaling at least in male fish. However, the authors highlight the fact that this may be a strain-specific result and, therefore performing this experiment in the inbred GRZ strain may be of interest (Müller et al., 2025).

#### 1.6.4 Olfaction and gustatory responses

Modulating olfactory and gustatory chemical signals has been reported to alter lifespan in *C. elegans* and *D. melanogaster* (Matei et al., 2021; Alcedo and Kenyon, 2004). While olfactory epithelium and taste bud morphology were recently characterized in adult MZM-0410 *N. furzeri*, transcriptomic data revealed limited detection of gustatory- and olfactory-related genes across several ages (Baumgart et al., 2014; Giaquinto et al., 2024). However, the transcriptome was assembled from brain tissue, which may not have included olfactory epithelium and would be free from tissues in which taste buds are located, such as the epithelium lining the lips, mouth, pharynx, oesophagus and gill (Baumgart et al., 2014).

#### 1.6.5 Neuropeptides

In vertebrates, orexin A (OXA) and neuropeptide Y (NPY) are hypothalamic neuropeptides that regulate physiological processes including feeding behaviour (Soya and Sakurai, 2020; Loh et al., 2015; Bouâouda and Jha, 2023). Impaired OXA and NPY signalling has been linked to age-related disorders and diseases, such as obesity and neurodegeneration (Nixon et al., 2015; Ferreira-Marques et al., 2025). An increase in fat deposition is observed at old age in the GRZ strain, and this could be linked to a dysregulation of feeding behaviour due to changes in OXA and NPY expression with age (Ripa et al., 2023b). RT-qPCR data and RNA-seq data analysis indicate that age does not alter the expression levels of *Hcrt* (encodes OXA) in whole brains isolated from MZM-0410 males. However, the authors point out that the absence of differences may be due to the ages selected for sampling, and that including samples from older fish may reveal differences in *Hcrt* levels compared to adults and young fish. The recently generated *Hcrt* knock-in *N. furzeri* will help to study *Hcrt* cell-type-specific expression (Bedbrook et al., 2023). In contrast, expression of *Npy* significantly increased with age (Montesano et al., 2019; Giaquinto et al., 2022). *Npy* expression has been reported to decrease in the hypothalamus yet increase in the striatum, medulla oblongata and pons in rodent models, even if protein levels decrease (Ferreira-Marques et al., 2025; Higuchi et al., 1991). Therefore, to better appreciate the role of NPY in brain aging, it may be necessary to focus on precise brain regions as well as compare expression levels to protein levels. NPY has also been reported to have a role in autophagy induction and that NPY overexpression in rats increased their lifespan (Michalkiewicz et al., 2003; Aveleira et al., 2015). Furthermore, NPY expression levels are decreased with age in rodents and in individuals with neurodegenerative diseases (Botelho et al., 2015). It will be important to explore the link between NPY and autophagy levels in the *N. furzeri* to determine if there is a potential impact on lifespan.

### 1.7 Mitochondrial dysfunction

With aging, mitochondrial function gradually deteriorates due to interrelated mechanisms such as the accumulation of mtDNA mutations, disturbances in proteostasis and changes in mitochondrial dynamics. This situation reduces the contribution of mitochondria to cellular energy production while increasing the level of ROS. If allowed to accumulate, ROS can trigger molecular damage and inflammation, leading to cell death (Srivastava, 2017). As a result, progressive deterioration in mitochondrial function contributes significantly to the aging phenotype.

#### 1.7.1 Mitochondrial membrane phospholipid composition

The mitochondrial membrane phospholipid composition of *N. furzeri* significantly changes with age. For example, the total phospholipid content of the mitochondrial membrane, as well as the proportion of phosphatidylcholine relative to total phospholipid, is reduced in older compared to younger MZM-0410 fish. In contrast, the ratios of phosphatidylserine, cardiolipin and phosphatidylethanolamine increased significantly, especially between 10 and 20 wph which coincides with a phase of rapid growth in the fish, suggesting a possible link between growth and changes in membrane lipid composition. The fatty acid composition of mitochondrial membrane phospholipids also changes significantly with age, which may reflect an accumulation of oxidative damage in mitochondrial lipids during the aging process (Almaida-Pagan et al., 2019). It has been suggested that these age-related changes in lipid composition contribute to mitochondrial dysfunction in *N. furzeri* by reducing membrane flexibility. Similar alterations have also been observed in aging mammalian brains, including mice and humans (Hong et al., 2016; Paradies et al., 2011). However, since the experiment using the *N. furzeri* relied on whole-body homogenates, these results may reflect changes in abundant tissues like skin or muscle. Due to the small size of *N. furzeri*, tissue-specific analysis remains technically challenging. Interestingly, the peroxidation index, which reflects the degree of polyunsaturation in mitochondrial membrane phospholipids, was significantly lower in *N. furzeri* compared to the related species *Nothobranchius rachovii*, which lives approximately twice as long. Since the “membrane pacemaker” theory links high membrane unsaturation with shorter lifespan, these findings appear to contradict the theory and rather suggests that the relationship between membrane composition and lifespan may be more complex than previously believed (Almaida-Pagan et al., 2019).

#### 1.7.2 Mitochondria-related gene expression

RNA sequencing of brain samples collected from MZM-0410 *N. furzeri* at five different ages revealed that numerous genes associated with mitochondrial structure and function were downregulated in older individuals (Baumgart et al., 2014). In old GRZ kidneys, a downregulation of mitochondria and energy-related pathways was noted in single-nucleus transcriptomic analysis (preprint Paulmann et al., 2025). The age-related decline in the expression of nuclear genes maintaining mitochondrial function may not only contribute to reduced cellular energy production and metabolic activity but may also indicate that mitonuclear interactions represent an evolutionarily conserved molecular marker of aging that underlies the short lifespan phenotype of *N. furzeri.*

Comprehensive genomic analyses comparing short-lived *Nothobranchius* species with longer-lived related species have revealed a positive selection in genes controlling mitochondrial biogenesis and energy production processes, including mtDNA replication and transcription, mitochondrial RNA processing and stabilization, mitochondrial protein translation, and the assembly of electron transport chain complexes (Sahm et al., 2017). Furthermore, some mitochondrial regulatory genes identified as being under positive selection in *N. furzeri* show similar evolutionary adaptations in mammals with unusually long lifespans, such as bats and blind mole-rats. Indeed, it has been demonstrated that interventions that disrupt or rearrange the balance between the mitochondrial and nuclear genomes in model organisms can affect lifespan (Sahm et al., 2017).

#### 1.7.3 mtDNA copy number and respiratory complexes

Despite the short lifespan of *N. furzeri*, significant deterioration in mtDNA copy number and mitochondrial function occurs in aging individuals; mtDNA copy number is significantly reduced in several tissues of old MZM-0403 fish compared to young fish (Hartmann et al., 2011). This decrease was consistent with the observed decline in the expression of the *Pgc1α* gene, one of the key regulators of mitochondrial biogenesis, as well as in the levels of *Tfam* and *Ssbp*, which are critical for mtDNA replication. These findings validate transcriptomic results, where a broader age-related downregulation of nuclear-encoded mitochondrial genes was observed in the brain of *N. furzeri* (Baumgart et al., 2014).

Functional alterations have also been reported alongside the reduction in mtDNA copy number and mitochondrial gene expression. In skeletal muscle, protein levels of Complex III and Complex IV of the respiratory chain were reduced in old fish, and a significant decrease in ADP-stimulated, succinate-driven respiration was reported (Hartmann et al., 2011). In another study, Complex IV and ATP synthase displayed stoichiometric losses in the brain of old fish (Kelmer Sacramento et al., 2020). In addition, imbalances in mitochondrial ribosome subunits were detected, and this was considered as a factor that may disrupt both protein synthesis and assembly of respiratory chain complexes. With the decrease in proteasome activity, protein aggregates including mitochondrial components accumulated and contributed to the mitochondrial dysfunction observed in the aged brain. These findings suggest that aging affects mitochondria not only at the transcriptional level but also in post-translational mechanisms of editing and repair.

Longitudinal lifespan analyses in *N. furzeri* have shown that high expression of genes encoding respiratory chain Complex I subunits was associated with shorter lifespan (Baumgart et al., 2016). This observation has raised the idea that a partial reduction in mitochondrial activity may have a protective effect. To test this hypothesis, the activity of Complex I was partially inhibited with rotenone, which led to a 15% increase in average lifespan of the exposed *N. furzeri.* Mild suppression of Complex I was also linked to a reversal of age-related regulation of gene expression. Notably, this effect was dose-dependent and exposure to higher levels of rotenone negatively impacted lifespan, supporting the idea of the protective effect of mitochondrial hormesis (Baumgart et al., 2016). In conclusion, pharmacological manipulation of Complex I levels in the *N. furzeri* model confirms the central role of mitochondrial function in aging and highlights the life-extending potential of mitochondrial-targeted interventions.

### 1.8 Cellular senescence

Cellular senescence occurs in response to cellular stress and/or damage, where cells will first undergo cell cycle arrest and second be cleared by immune cells. Impairment of immune clearance, occurring with age, induces pathogenicity, leading to accumulation of senescent cells, to fibrotic problems and to senescence-associated secretory phenotype (SASP) (Ovadya et al., 2018). Senescent cells are defined by several features, including lysosomal expansion, cell cycle arrest, altered nuclear morphology, the appearance of senescence-associated heterochromatin foci (SAHFs), and an increase in ROS and SASP factors. Indeed, SASP will turn senescent cells into proinflammatory cells by triggering the secretion of chemokines, cytokines, TGF-β, pro-fibrotic factors, matrix metalloproteases and/or growth factors (Coppé et al., 2010).

#### 1.8.1 Senescent markers

Histological markers of senescence were some of the first age-related markers measured in *N. furzeri*. Levels of lipofuscin autofluorescence and SA-β-Gal activity in liver samples were reduced in GRZ fish that were housed in aquariums maintained at 22 °C compared to 25 °C (Valenzano et al., 2006a). Lowering temperature is a well-known course of action to slow aging phenotypes (Conti and Hansen, 2013) and fish kept at 22 °C had longer lifespans as well as improved learning scores in an active avoidance task that indicated healthier brain aging (Valenzano et al., 2006a). SA-β-Gal activity and gene expression of several cell cycle inhibitors were increased in cells isolated from head kidney, skin and telencephalon of old *N. furzeri* individuals (Genade et al., 2005; preprint Morabito et al., 2023; Graf et al., 2013; Van houcke et al., 2023). Increased gene expression was supported by increased protein levels of “cellular senescence” KEGG pathway in proteomic analysis of the telencephalon (Van houcke et al., 2023). SA-β-Gal staining was also increased in various GRZ tissues at old age (i.e., liver, brain, intestine, kidney, heart, skeletal muscle and gonad), although the signal intensity was variable between organs and life stages with strong signals already occurring at newborn stages for some organs, possibly showcasing the protective function of senescence (Schöfer et al., 2024).

KEGG analysis from RNA-seq data performed on muscle tissue from MZM-0410 young, adult, and old fish revealed an age-related signature of senescence. RT-qPCR analysis confirmed specific expression of genes related to immune, inflammatory response and senescence. Moreover, markers of senescence and SASP were observed with age in skeletal muscle, including changes to nuclear morphology, cytokine secretion, and formation of SAHFs (Cencioni et al., 2019). In addition, senescent cells are known to accumulate impaired mitochondria (Fang et al., 2016) and skeletal muscle samples from old fish had reduced numbers of mtDNA and decreased expression of genes associated with mitochondrial biogenesis (Cencioni et al., 2019). Most recently, markers of cellular senescence were measured in the aging heart of GRZ *N. furzeri*. In addition to increased SA-β-gal staining, the hearts from older individuals displayed increased expression of genes related to SASP, cell cycle inhibition and DNA damage (Ma et al., 2025).

Several other transcriptomic studies demonstrated a role of cellular senescence in *N. furzeri* aging. A data set including four tissues from four different vertebrates (*N. furzeri*, *D. rerio*, *M. musculus*, *H. sapiens*) was analysed to identify similarities between age-regulated processes and disease signature. Cell cycle mechanisms were downregulated with age (Aramillo Irizar et al., 2018). “Cell cycle” is also one of the five common KEGG pathways downregulated in several tissues of *N. furzeri* with age (Baumgart et al., 2016). Gene enrichment analysis of RNA-seq data was studied and differentially expressed genes were grouped according to the expression patterns across ages. Genes that were found to peak at 27 wph included those whose protein products inhibit the cell cycle, in contrast the lowest expressed genes at this age were those whose proteins positively regulate cell cycle and DNA replication, supporting a role of cellular senescence in aging (Baumgart et al., 2014; Evert et al., 2003). Finally, in a longitudinal fin clip analysis, fish at 10 and 20 wph show a downregulation of “cell cycle” KEGG pathway with age in shorter-lived fish. From this study, senescence can be considered as a mortality risk factor and as a contributor of aging (Kelmer Sacramento et al., 2020). Age-related fibrosis in the intervertebral discs of older fish has also been observed, suggesting impaired regulation of immune clearance within the senescence pathway, leading to fibrotic problems (Butylina et al., 2023).

A transparent KO line of *N. furzeri* (MZCS-08/122), *klara*, was successfully generated in 2023. Expressing GFP into the locus of a cell cycle inhibitor to generate a functional senescence reporter, the authors detected an increase in GFP-positive cells in the liver of old fish compared to young fish of this new KI line (Krug et al., 2023). To determine if the cells are truly senescent, it will be necessary to measure several senescent-related markers, such as those mentioned above.

#### 1.8.2 *In vitro* cell senescence

In contrast to *in vivo* tissues, neither embryonic cell lines or cultured fibroblasts from young and old *N. furzeri* appear to undergo senescence with advanced passages (Graf et al., 2013; Součková et al., 2023). The absence of cell senescence has also been observed in other cell lines derived from fish species and rodents (Komura et al., 1988; Vo et al., 2015; Seluanov et al., 2008). In contrast, γ-irradiated primary fin fibroblasts led to increased SA-β-Gal activity, something that was detected at lower levels in fibroblasts isolated from fin samples of *cGas* KO fish that are characterized by reduced innate immune signaling (preprint Ballhysa et al., 2024). Therefore, while the *in vitro* situation may not help to fully understand the role of cellular senescence in *N. furzeri*, the use of radiation could help to better understand the links between senescence and immune clearance. Also, other cell lines like neuronal or stem cell could reveal a different impact of aging on their putative senescence.

#### 1.8.3 Senolytics

Treatment to remove senescent cells, such as with the use of senolytics revealed promising results in *N. furzeri*. Intraperitoneal injections of dasatinib and quercetin (D/Q) significantly reduced SA-β-Gal staining within the telencephalon compared to vehicle treated fish. This was accompanied by significantly lower senescence-associated gene expression levels. Furthermore, an increase in TUNEL labelling suggest that the D/Q treatment induced apoptosis of senescent cells (Van houcke et al., 2023). D/Q treatment through oral gavage similarly reduced senescent markers in heart tissue of old fish (Ma et al., 2025). Fisetin treatment of head kidney cells isolated from old fish also improved the immune response to levels that mirrored those seen in untreated young cells (preprint Morabito et al., 2023). D/Q and fisetin treatments have previously been found to increase lifespan in other animal models and improve health in clinical trials (Hickson et al., 2019; Justice et al., 2019; Yousefzadeh et al., 2018). The use of *klara* fish or *cGas* KO fish combined with senolytics treatment could help with the discovery of mechanisms linked to fibrotic diseases or age-related diseases linked to senescence.

### 1.9 Stem cell exhaustion

Stem cells have the remarkable ability for self-renewal, however the progressive decline in function and pool size has been implicated in reduced age-related tissue repair and regeneration (Rando et al., 2025).

#### 1.9.1 Stem cell niches

While neural stem cell niches in mammals are limited to the telencephalon (or cerebrum in humans), stem cell niches in teleost fish are distributed along the entire rostro-caudal extent of the ventricular surface. However, like in mammalian brains, adult neurogenesis declines with advanced age in *N. furzeri* brains (Kase et al., 2020; Tozzini et al., 2012). Neuronal cell proliferation was reduced in the brains of old MZM-0410 fish compared to young fish assessed by EdU positive staining in tissue sections. Additionally, reactivity of doublecortin, a protein necessary for adult neurogenesis in mammals, was also reduced in the aged brains of *N. furzeri.* MicroRNAs play dynamic roles in neurogenesis, and 165 conserved miRNAs have been detected in the brain of *N. furzeri* (Baumgart et al., 2012). Expression of miRNAs has been carried out using several approaches, for example, qPCR, miRNA-seq and *in situ* hybridization results indicate an age-dependent upregulation of miR-15a, and downregulation of miR-20a and the miR-17–92 micro cluster, which encodes for 15 miRNAs in MZM-0410 *N. furzeri* (Baumgart et al., 2012; Terzibasi Tozzini et al., 2014). miR-15a is a negative regulator of the cell cycle and its increased detection in the neurogenic niches of the telencephalon and optic tectum of old brains compared to young brains supports an age-related decrease in cell proliferation (Terzibasi Tozzini et al., 2014). In GRZ aged gut, cell cycle dynamics of intestinal stem cells (ISCs) were reduced and RNA-seq analysis revealed changes in markers of ISCs and stem cell niche factors, indicating a decreased capacity for tissue regeneration with age (Kothmayer et al., 2025).

Coregulatory gene networks were created using RNA-seq data from whole brain of MZM-0410 *N. furzeri* at several different ages. Among the genes that were differentially expressed with age, the putative transcription factor zinc-finger protein 367 (*Znf367*), which appears to be the central hub in a network enriched for genes associated with cell cycle regulation, was identified (Baumgart et al., 2014). *In situ* hybridization and immunohistochemistry were used to confirm the age-related decrease in *Znf367* gene expression in the optic tectum of MZM-0410 *N. furzeri*. While evidence suggested reduced cell proliferation in the optic tectum, several other genes not previously associated with neurogenesis in other vertebrate models were downregulated, suggesting that *N. furzeri* could reveal potential novel targets for stem cell research. RNA-seq data from two independent studies explored whole brain age-related changes in family members of the Notch signaling pathway, which is involved in the control of neurogenesis and cell fate during embryonic development (Gonzalez and Reinberg, 2025). Analysis of the two MZM-0410 transcriptomes revealed a steep decline in several Notch family members (Bagnoli and Terzibasi, 2021).

#### 1.9.2 Progenitor cells

The recent characterization of neurogenesis in *N. furzeri* revealed two types of progenitor cells: radial glial and non-glial progenitors, the latter accounting for the majority of proliferating progenitors in the telencephalon of this fish. Radial glial precociously transition to a quiescent state whereas non-glial progenitors appear to be primarily responsible for driving neuro-differentiation in the rapidly growing juvenile dorsal telencephalon (Zandecki et al., 2025; Coolen et al., 2020; Ayana et al., 2024). Telencephalons isolated from young and old female GRZ *N. furzeri* failed to display significant changes in the cell type due to aging. However, there were signs of reduced proliferative capacity in the non-glial progenitor cells. In addition, transcriptional profiles of the non-glial progenitor pools revealed an age-dependent upregulation of genes associated with inflammation (Ayana et al., 2024). In a separate cohort of fish, while the small percentage of dividing radial glial remained relatively unchanged between young and aged female telencephalon, the dividing non-glial progenitors significantly declined with age. This was observed in naïve brains as well as within brains subjected to stab-wound injuries. These results highlight both overall reduced adult neurogenesis and reduced capacity for regeneration in the adult telencephalon (Van houcke et al., 2021). Intraperitoneal D/Q injection reinstated neurogenesis in the brain of aged fish following traumatic brain injury, specifically the total number of proliferating nonglial progenitors was significantly upregulated compared to those in the brain of vehicle injected fish, highlighting the potential of senolytics not only for healthy aging but also for regeneration in the aging brain. Wound healing has also been explored in response to caudal fin amputation (Wendler et al., 2015; Örling et al., 2023; Ortega Granillo et al., 2024). In two independent studies, the rate and the zone of fin regeneration was reduced with age in both MZM-0703 and MZCS-222 strains, when measured up to 10 days post amputation (Wendler et al., 2015; Örling et al., 2023). While studies are still needed to verify the role of dedifferentiation and transdifferentiation in fin regeneration, the current evidence suggests that the progenitor cells have a role to play in fin regeneration and that this pool is reduced in fins from older individuals.

These results suggest that with increased age, stem cell regulation is impaired and could have negative effects on self-renewal, differentiation, and maintenance in a quiescent or active state. The use of *N. furzeri* can provide us with an opportunity to explore regenerative medicine that can restore repair capacity in aging tissues.

### 1.10 Altered intercellular communication

Age-related changes in intercellular communication can affect hormonal and/or neural signalling factors. Soluble factors (i.e., cytokines, ligands) and short-lived molecules (i.e., ROS, prostaglandins, nucleic acids) are involved in intercellular signalling through cell receptors or directly between neighbouring cells using various cell junctions. Determining whether levels of these factors change with age may provide a novel target to improve health at later ages (Miller et al., 2020; Fafián-Labora and O’Loghlen, 2020).

#### 1.10.1 Long- and short-range communication systems

The impact of long- and short-range communication systems in *N. furzeri* aging has largely been overlooked. However, the GO term “gap junction” is increased with brain aging in *N. furzeri* (Van houcke et al., 2023). In addition, the longitudinal fin RNA-seq analysis identifies the KEGG term “tight junction” as a mortality risk factor with increased age (Kelmer Sacramento et al., 2020). Gap and tight junctions are directly implicated in cell-to-cell communication, but further studies are necessary to understand why they increase with age and whether they have a role in aging. A potential limitation to explore this hallmark in *N. furzeri* is the difficulty of (multi)sampling. While repeated blood sampling on live *N. furzeri* is feasible, there is no current study exploring anti- or pro-aging blood borne factors (Dolfi et al., 2023). This may be due to low collection recovery, but zebrafish have been used successfully for this purpose (Pedroso et al., 2012).

A recent preprint evaluated the number of ligand-receptor interactions between renal and vascular cell types. A reduction of these interactions, indicating reduced cell-cell adhesion, was reported in old GRZ kidneys, where nephrosclerosis, microvascular rarefaction, and glomerular dysfunction occurs. Inhibition of sodium-glucose linked transporter 2 (SGLT2) by dapagliflozin, a treatment used to improve renal function in humans, was administered through the diet to *N. furzeri*. Treated fish had a partial restoration of glomerular function and an increase in ligand-receptors interactions, independent to lifespan extension, indicating a mitigation of age-related kidney dysfunction (preprint Paulmann et al., 2025).

Dabrowski et al. (2020) developed a method to quantify steroids and sterols within gut, liver, gonads and brain tissue of GRZ *N. furzeri* (Dabrowski et al., 2020). Progesterone and testosterone were significantly decreased in the gonads with age as expected for sex steroid hormones. Intermediates of cholesterol synthesis were significantly reduced in all tissues except the gut. Due to the central role of cholesterol in steroid synthesis, its reduced biosynthesis could affect many biological functions with age. 24-hydroxysterol is decreased in *N. furzeri* brains as seen in Huntington’s and Alzheimer’s disease (AD) patients while 27-hydroxysterol is increased as in AD and atherosclerosis patients (Kreilaus et al., 2016; Brown and Jessup, 1999; Wu et al., 2022). Together those sterols could be used as relevant biomarkers in age-related diseases, especially with the idea that brain aging in *N. furzeri* mimics neurodegeneration rather than healthy human brain aging (de Bakker and Valenzano, 2023).

Other potential neuronal biomarkers could include inhibitors of DNA binding proteins (Id), which are transcription factors that regulate cellular differentiation and proliferation in neurogenic processes. Gene levels of *Id3* were increased within old MZM-0410 brains, showcasing possible changes in neurogenic processes with age (Leggieri et al., 2021). Nerve growth factor and tyrosine kinase receptors interact with neurotrophins that are implicated in neuronal cell survival, neurite outgrowth, and neuronal differentiation. The distribution of receptors and neurotrophins is well characterized in the *N. furzeri* brain and age does not impact their expression and localization (D’Angelo et al., 2014a; D’Angelo et al., 2016; Leggieri et al., 2019; de Girolamo et al., 2020; D’Angelo et al., 2014b). As neurotrophins are implicated in general brain neuronal maintenance, their continued study in relation to aging and neurodegenerative diseases could be of relevant interest. Additionally, the Klotho gene family includes secreted forms released into the blood, urine, and cerebrospinal fluid. Rodent studies suggest a role for *αKlotho* in lifespan regulation and a tumor suppressor role for *βKlotho* (Kurosu et al., 2005; Ye et al., 2013). *αKlotho* expression increased in an age-dependent manner in the liver of GRZ fish, whereas *βKlotho* levels decreased (Zupkovitz et al., 2018b). Low levels of *βklotho* support the purported neoplastic lesions detected in liver of old fish (Di Cicco et al., 2011; Baumgart et al., 2015).

#### 1.10.2 Extracellular matrix

The extracellular matrix (ECM) is responsible for providing structural support to tissues. Its dysregulation with age can affect intercellular communication. Of note, tissue fibrosis, can be caused by several defects in ECM components (Selman and Pardo, 2021). RNA-seq data reveals a downregulation of genes related to the ECM in both skeletal muscle and brain of older *N. furzeri* fish, suggesting a remodeling of the ECM during aging (Baumgart et al., 2014; Cencioni et al., 2019). In skin biopsies from MZM-0403 and GRZ fish, a downregulation of “collagen-/proteoglycan-related” genes localized in the “extracellular region” was also reported (Petzold et al., 2013). These results were confirmed by measuring expression of specific genes related to collagen and/or the ECM (Baumgart et al., 2015; Gu et al., 2014). Similarly, several collagen genes were reduced with age in MZM-0410 heart (Heid et al., 2017). Interestingly, knockdown of ECM genes has been linked to lifespan extension in *D. melanogaster* heart (Sessions et al., 2017). These results may be gene- or tissue-dependent or suggest that lifelong reduction of ECM genes is beneficial compared to a gradual age-related loss. In contrast, the expression of some collagen-related genes increased during brain aging in MZM-0410 fish. Parallel to this, an increase in markers of gliosis was noted, confirming signs of neurodegeneration with age in *N. furzeri* (Leggieri et al., 2022). Noteworthy, age-related increases or transgenic overexpression of specific collagen-related genes have been linked to stroke and vascular dementia in humans and in AD-like phenotypes in mice, respectively (Uspenskaia et al., 2004; Tong et al., 2010). Indeed, collagen accumulation is a preliminary step in the occurrence of fibrotic diseases and begins through the accumulation of ROS that induce TGF-β that in turn activate fibroblast and collagen accumulation (Liu and Desai, 2015). In this light, *N. furzeri* could be a good model to study mechanisms counteracting fibrotic diseases.

### 1.11 Chronic inflammation

Age-related chronic inflammation (inflammaging) is characterized by the low activation of the immune system, even in the absence of infection or injury, resulting in elevated levels of inflammatory markers (Franceschi et al., 2000). Inflammaging has been linked to several age-related diseases and specifically, the pro-inflammatory cytokine, IL-6 has been linked to increased mortality risk in humans (Hirata et al., 2020).

#### 1.11.1 Inflammatory-related genes and proteins

Transcriptome analysis revealed that genes linked to inflammation were upregulated in skeletal muscle isolated from old male MZM-0410 *N. furzeri* compared to young fish. Individual gene measurements confirmed these results, indicating age-related increases in immune and inflammatory response genes (Cencioni et al., 2019). Similar results were reported in transcriptomic studies that included kidney, liver, brain, skin and blood samples, with a clear age-related induction of genes involved in immunity and inflammation (preprint Paulmann et al., 2025; Aramillo Irizar et al., 2018; Petzold et al., 2013). Single-nucleus RNA sequencing of kidney tissue from MZM-0410 fish treated with dapagliflozin revealed an attenuation of pro-inflammatory gene expression, indicating reduced inflammaging (preprint Paulmann et al., 2025). More recently, transcriptomic analysis revealed an age-related increase in inflammatory-related genes within brain, heart, skeletal muscle and spleen isolated from male and female GRZ fish (Xu et al., 2023). The GRZ transcriptome of intestinal tissue from old fish was enriched for genes within the GO terms linked to inflammation and immunity (Smith et al., 2017). Several inflammatory genes were also reported upregulated in the adipose tissue of old GRZ fish compared to young individuals. Using a pan-leukocyte marker, immunoreactive cells were detected at higher numbers in adipose tissue, heart and skeletal muscle of old, fasted fish compared to old, fed fish, showcasing the beneficial effect of caloric restriction (discussed above) on the activity of immunity (Ripa et al., 2023a).

Using a broad “omics” approach, authors of a recent preprint revealed that plasma from old GRZ *N. furzeri* was enriched in proteins that grouped to proteolysis, coagulation, nucleosome structure and apoptosis GO terms (preprint Morabito et al., 2023). However, no pro-inflammatory cytokine protein peptides were detected, instead there were age-related increases in complement proteins and coagulation factors that are associated with the inflammatory response in other teleost fishes. The authors expanded their search and noted age-related fibrosis in the head kidney. Proteomic analysis of plasma and head kidney revealed that components of DNA repair machinery decreased whereas pro-inflammatory proteins increased with age (preprint Morabito et al., 2023). These proteomic changes correlated with an impaired immune response within immune cells extracted from the head kidney of old fish challenged with LPS. Reduced immune function may be due to the decline in antibody repertoire diversity within the *N. furzeri* that has been noted at the level of the intestine and the whole body (Bradshaw et al., 2022). Furthermore, the age-dependent decline in DNA repair machinery correlated to higher levels of senescent immune cells. As such, a pre-treatment with the senolytic, fisetin, recovered the immune response of the old fish to levels comparable to young fish. These results were specific to immune cells recovered from old fish, as there were no changes in the immune response between untreated and treated immune cells from young fish. It will be interesting to determine if this treatment alters the pro-inflammatory proteome from within these cells and whether an *in vivo* treatment also improves the immune response in old fish.

#### 1.11.2 cGAS/STING pathway

The cGAS/STING pathway, which has a role in innate immunity, was recently reported to be conserved within the *N. furzeri*. With a focus on the kidney and gut, two tissues associated with immune response, loss of *cGas* function through CRISPR-Cas9 resulted in a renal transcriptome that suggested increased inflammation, whereas loss of STING led to limited differences in gene expression in both organs. Following irradiation, genes involved in inflammation were upregulated in the kidneys of *Sting* KO fish compared to WT, a finding that differed from *cGas* KO fish (preprint Ballhysa et al., 2024). These results suggest the involvement of STING against insult-induced inflammation.

#### 1.11.3 Inflammatory response to injuries

Inflammatory markers following a stab wound injury, revealed that inflammation occurred at higher levels in aged GRZ brains compared to young female fish. This included increased levels of L-plastin reactivity indicating more microglia/macrophages as well as elevated expression levels of colony-stimulating factor 1 receptor (*Csfra1)* and pro-inflammatory *IL-8*. Furthermore, in young brains, the expression levels of these genes dropped to pre-injury levels by 9 days post injury, whereas this was not the case in old brains, reaching baseline levels by 23 days post injury. Although not significant, in naïve brains the levels of L-plastin positive cells and gene expression levels are elevated in the old animals compared to the younger ones, already indicating increased inflammation in the brain. Injury increased *L-plastin* and *Csf1ra* gene expression in both young and old fish to approximately the same response (Van houcke et al., 2021). Similarly, optic nerve crush was associated with higher levels of inflammatory markers (L-plastin; microglia/macrophage and vimentin; reactive gliosis) in older *N. furzeri* GRZ female fish retina and optic tectum. These levels were already elevated in the retina and the optic tectum of naïve older fish when compared to younger fish (Vanhunsel et al., 2021; Vanhunsel et al., 2022). Activated gliosis assessed by glial fibrillary acid protein (GFAP) staining was also increased in older fish 7 days post injury, along with a select measurement of genes involved in inflammation. Taken together, these data suggest that a pro-inflammatory pathway is favored with increased age, and the elevated immune response may limit the rate of repair (Vanhunsel et al., 2021). These results were supported by evidence of age-related gliosis, noted by increased GFAP reactivity and GFAP expression observed in optic tectum sections and whole brain of MZM-0410 *N. furzeri* (Tozzini et al., 2012).

The specific transcription factors, STAT3, STAT5.1, that regulate chronic inflammation have been located within the genomic region that is associated with lifespan differences between GRZ and MZM *N. furzeri* (Valenzano et al., 2015). Creating mutant fish for these transcription factors together with the use of existing *cGas* or *Sting* KO fish, could help to reveal the impact of immunity decline in lifespan. Several anti-inflammatory drugs are under clinical trials with some providing promising results on several age-related diseases (i.e., canakinumab) (Ridker et al., 2017) while others are contradictory (i.e., aspirin) (McNeil et al., 2018). *Nothobranchius furzeri* could be a practical platform to further test these drugs and other new anti-inflammatory drugs (like vedaprofen) to decipher their mechanisms of action on inflammation and microbiota as well as their effects on lifespan.

### 1.12 Dysbiosis

The gut microbiome and its dysfunction with age have increased in interest over the last decade (Pezzino et al., 2024). Although a general decline in the gut bacterial diversity seems to occur with age, no clear consensus exists on the taxa that promotes healthy aging (Van Hul et al., 2024).

#### 1.12.1 Microbiota alterations

When studying 16s RNA gene amplicon in young and old *N. furzeri*, a significant reduction of bacterial taxonomic diversity was found, while the bacterial abundance was unchanged (Smith et al., 2017). Young fish gut microbiota was enriched with bacteria associated with glycolysis, polysaccharide metabolism and DNA repair while old fish gut microbiota was enriched with bacteria associated with pathogenesis. In parallel, upregulated genes in young fish intestine were associated to cell cycle activity (i.e., proliferation and differentiation) while for old fish intestine, they were grouped by GO terms linked to immune and defense responses against pathogens and inflammation. The gut composition, transcriptome data of the host, and metagenome analysis of the microbiota clearly suggest that young fish have a healthy gut better able to preserve homeostasis while old fish have an unhealthier and likely pathological gut (Smith et al., 2017).

#### 1.12.2 Gut microbiota transfer

While studies have been carried out exploring microbiota transplant in mice (Bárcena et al., 2019; Xu et al., 2022), Smith *et al.* were the first team demonstrating effects of gut content transplantation on aging in a vertebrate model, with clear signs that transplanting the gut content from young to old individuals improved signs of health and extended lifespan (Smith et al., 2017). Additionally, this type of transplant resulted in differential expression in genes associated with the TOR pathway, cell adhesion and extracellular matrix composition, suggesting their importance for improving healthspan and lifespan. Seeing the success and unprecedented discoveries with a gut content transplant, *N. furzeri* could be easily used to test the effect of probiotic therapies or the use of fecal microbiota transplant, both having positive outcomes on the longevity of different mice models and showing encouraging signs of healthier aging in humans (Ghosh et al., 2022).

## 2 Discussion

As highlighted in this review, *N. furzeri* show alterations occurring with age that are comparable with human aging for almost all of the twelve hallmarks. While complementary studies are needed to draw better parallels between humans and *N. furzeri* for some hallmarks (i.e., mtDNA mutations, nuclear architecture, telomerase activity, anti- and pro-aging blood borne factors, etc.), only one specific hallmark is clearly characterized by opposite changes with age compared to humans: disabled macroautophagy. For example, expression of autophagy-related genes is decreased with age in humans, whereas they are upregulated in *N. furzeri*. Moreover, autophagy was induced in several model organisms and led to an extension of lifespan or/and healthspan. Whether the natural induction of autophagy seen in *N. furzeri* is responsible for healthier aging within their short lifespan is unclear. Overexpressing or repressing autophagy-related genes along with lifespan measurements in *N. furzeri* would help to better understand the links between autophagy and good health in the fish but also in humans. Although advantages and weaknesses of *N. furzeri* model have largely been discussed in previous reviews (Genade and Wilcox, 2021; Holtze et al., 2021; Platzer and Englert, 2016; Kim et al., 2016), we want to highlight that using *N. furzeri* have allowed for new discoveries, such as the existence of several unstudied aggregating proteins, the role of the Ire-Xbp1 axis in skin aging, and the use of microbial transfer for healthier gut aging.

Interventions to accelerate aging in *N. furzeri* have been tested for telomere attrition, loss of proteostasis, disabled autophagy, mitochondrial dysfunction, chronic inflammation hallmarks while interventions to decelerate aging in *N. furzeri* have been tested for loss of proteostasis, deregulated nutrient-sensing, mitochondrial dysfunction, cellular senescence, altered intercellular communication, chronic inflammation and dysbiosis hallmarks. When an increase in lifespan is seen, it is often accompanied by positive health effects reinforcing that these interventions have an impact not only on aging but also on health and that they do not widen the gap between healthspan and lifespan, rather they shorten it. As highlighted in Table 1, there is room to further use *N. furzeri* to establish those interventions on other hallmarks and rapidly see clear impacts on lifespan that require more time in other vertebrate models. To increase our knowledge on aging, it is necessary to fill some gaps but, some consensus is also needed to contribute to aging research in an efficient way.

First, the use of different strains that are considered as short- and long-lived can be seen as a double-edged sword. On one side, it can complicate comparison between studies due to differences in genome and lifespan. In this regard, each facility should record a lifespan curve and appropriately choose young, adult and old timepoints. From this review, we can highlight that for GRZ; young fish range between 5–7 weeks, adult between 8–14 weeks and old fish between 15–22 weeks; whereas for MZM, young fish are considered between 5–8 weeks, adult between 9–24 weeks and old fish between 25–40 weeks. On the other side, strain specificity may aid our knowledge of what is underlying lifespan. For example, the GRZ strain experiences an anticipated aging profile compared to the MZM strain, which likely contributes to its shorter lifespan (Mazzetto et al., 2025). Valenzano et al. (2015) have identified age-related genes with non-synonymous single nucleotide polymorphism shared between several MZM strains but not with GRZ. They then classified these genes according to the nine original hallmarks of aging (López-Otín et al., 2013). Using genomic tools, the identified MZM genes and the GRZ aging profile represent an interesting starting point to study genes that could help to ameliorate healthy lifespan.

Creating transgenic lines is an essential gap in the field that is gradually being closed. While precise knock-in (KI) and KD procedures are emerging (Bedbrook et al., 2023; Kushawah et al., 2020), transgenesis protocols using *N. furzeri* have been available for a decade (Harel et al., 2015; Hartmann and Englert, 2012; Allard et al., 2013; Valenzano et al., 2011). In 2015, Harel and colleagues targeted genes specific to the hallmarks of aging and successfully generated six stable KO lines (Harel et al., 2015). Currently, several more KO lines have been added to the toolbox and are under use as shared at the *Nothobranchius* symposium 2025 (Jena, Germany). These lines encompass a variety of genes that have links with aging hallmarks, including genomic instability, epigenetic regulation, inflammation, autophagy and intercellular communication. Still there is a lack of fish lines with an overexpression of specific genes in those pathways.

Although two standardized breeding protocol have been published (Dodzian et al., 2018; Polačik et al., 2016), differences in animal care still occur between facilities, mainly at the level of tank size, male/female ratio, and diet. Yet these represent a third ongoing concern. Diet is one of the biggest differences in fish husbandry that could affect the robustness of aging studies. While most labs use live *Artemia* nauplii and frozen/live red mosquito larvae (bloodworms), their origin and composition can vary and largely impact caloric intake. As seen in the deregulated nutrient-sensing hallmark, calorie content can largely affect lifespan and in turn affect the validity of results. A pelleted diet that ensures dietary needs of the fish should be a priority and has already been tested (Žák et al., 2020). However, it is currently not commercially available. A pelleted diet would be a gold-standard to play on the composition in collaboration with the company, using different levels of therapeutic, macronutrients or micronutrients. Furthermore, the use of automated feeders has highlighted the cognitive decline observed in older fish, therefore it may be important to consider how the feeding schedule can impact age-related pathologies, possibly due to shifts in daily rhythms (McKay et al., 2022).

The possibility to properly administer a drug and making it available for the target is our fourth point of concern for aging studies. Several techniques are currently used, including intraperitoneal injection (Kelmer Sacramento et al., 2020; Van houcke et al., 2023; Tozzini et al., 2012), aqueous exposition (Baumgart et al., 2016), food consumption (preprint Paulmann et al., 2025, Valenzano et al., 2006b), and oral gavage (Ma et al., 2025). While precise needle injections can represent a stress affecting results, aqueous exposition may not ensure drug absorption (i.e., decomposition in water, short half-life, uptake quantity) and dietary intake can complicate the quantity of drug dosed. However, we are optimistic that these techniques can help to discover new avenues to decelerate aging using specific therapeutic.

Overall, *N. furzeri* represents a practical and valuable model for aging research. The short lifespan, ability to work in a high throughput setting and the resemblance to human aging will help to unravel new targets of interests in each hallmark of aging and facilitate the implementation of clinical studies on age-related diseases as various as Alzheimer’s disease, Parkinson’s disease, amyotrophic lateral sclerosis, frontotemporal dementia, fibrotic diseases, progeria disease as highlighted in this review.
